# Preparation and In Vitro Characterization of Microneedles Containing Inclusion Complexes Loaded with Progesterone

**DOI:** 10.3390/pharmaceutics15061765

**Published:** 2023-06-19

**Authors:** Hongji He, Zhaozhi Wang, Kadireya Aikelamu, Jingya Bai, Qi Shen, Xiaoli Gao, Mei Wang

**Affiliations:** College of Pharmacy, Xinjiang Medical University, Urumqi 830017, China

**Keywords:** microneedle, inclusion complex, progesterone, transdermal

## Abstract

Objective: In order to improve patient compliance and the ease of use during progesterone application, and to increase the clinical application of progesterone, progesterone was made into a microneedle. Methods: Progesterone complexes were prepared using a single-factor and central composite design. In the preparation of the microneedles, the tip loading rate was used as an evaluation index. The selection of tip materials among the biocompatible materials of gelatin (GEL), hyaluronic acid (HA), and polyvinylpyrrolidone (PVP), and the use of polyvinyl alcohol (PVA) and hydroxypropyl cellulose (HPC) as backing layers, respectively, were carried out and the resulting microneedles were evaluated accordingly. Results: The progesterone inclusion complexes prepared at a molar ratio of 1:2.16 progesterone and hydroxypropyl-β-cyclodextrin (HP-β-CD), a temperature of 50 °C, and reaction time of 4 h had high encapsulation and drug-loading capacities of 93.49% and 9.55%, respectively. Gelatine was finally chosen as the material for the preparation of the micro-needle tip based on the drug loading rate of the tip. Two types of microneedles were prepared: one with 7.5% GEL as the tip and 50% PVA as the backing layer, and one with 15% GEL as the tip and 5% HPC as the backing layer. The microneedles of both prescriptions exhibited good mechanical strength and penetrated the skin of rats. The needle tip loading rates were 49.13% for the 7.5% GEL-50% PVA microneedles and 29.31% for the 15% GEL-5% HPC microneedles. In addition, in vitro release and transdermal experiments were performed using both types of microneedles. Conclusion: The microneedles prepared in this study enhanced the in vitro transdermal amount of progesterone drug by releasing the drug from the microneedle tip into the subepidermis.

## 1. Introduction

Endogenous progesterone is primarily produced in the corpus luteum, ovaries, and placenta [1]. Progesterone primarily prevents and treats threatened abortion, preterm labor, anovulatory irregular menstruation, premenstrual tension syndrome, dysfunctional uterine hemorrhage, and amenorrhea [2,3,4]. The combination of oestrogen and progestin in late menopause has been shown to reduce the risk of endometrial thickening and endometrial cancer associated with the use of oestrogen alone [5]. The commonly used routes of administration are intramuscular, transvaginal, and oral [6,7,8,9]. Intramuscular progesterone is highly effective and inexpensive, and it is the traditional drug used for luteal support in assisted reproductive technology (ART) [10]. However, it has a high incidence of adverse reactions, may cause allergic reactions, is inconvenient to inject intramuscularly daily, and is prone to local nodules from pain and irritation at the injection site, as well as local aseptic abscesses and sciatic nerve injury [11,12]. When compared to intramuscular progesterone, vaginal progesterone has a higher incidence of luteal phase vaginal bleeding, low bioavailability, and other issues that limit its clinical application. 

The transdermal drug delivery system (TDDS) is a popular and emerging drug delivery system in recent years in pharmacy research [13,14]. It mainly refers to the drug administered through a specially designed formulation device in which the drug is introduced into the inner skin without damaging the skin, and then enters the human circulatory system to achieve the purpose of systemic drug administration [15]. Due to its characteristics of improving patient compliance, maintaining stable blood concentration, avoiding hepatic and intestinal first pass effects, and improving medication safety, it has become an important part of modern medicine and, at the same time, has a good development prospect [16,17,18].

The skin is the largest organ of our body; it is a chemical and physical barrier, a site of temperature regulation, and a terminal sensory organ [19]. Anatomically, the skin is divided into three parts: the epidermis, the dermis, and the subcutaneous tissue. Due to the presence of the skin barrier, most drugs cannot penetrate the skin and enter the bloodstream directly, making it difficult to obtain the same bioavailability as other agents [20].

Microneedles have sparked intense attention as a transdermal drug delivery technique owing to their unique three-dimensional microstructure, which can penetrate the cuticle and increase drug permeability and absorption [21,22]. Drug molecules can be delivered to the skin through microneedles in a minimally invasive manner, which has the advantages of being painless, promoting patient compliance, and facilitating self-administration [23]. It also addresses the limitations of conventional transdermal drug delivery, such as allergenicity and shallow delivery sites [24,25,26]. The first generation of solid microneedles for transdermal drug delivery are made of silicon, metal, and ceramic, which can overcome the skin barrier and enhance drug penetration [27,28]. However, the drug loading rate is low, the needle tip is easily dislodged, and the preparation process is complex and expensive. Soluble microneedles are made from biodegradable polymeric materials and drugs [29]. After the soluble microneedle has pierced the skin, the needle gradually dissolves or degrades in the microenvironment, releasing the drug at the same time [30]. Soluble microneedles have opened up new avenues of clinical drug delivery with their unique advantages of safety, efficiency, biocompatibility, and minimal trauma [31].

This study proposes to prepare a soluble microneedle loaded with progesterone. In this study, hyaluronic acid (HA), gelatin (GEL), polyvinyl alcohol (PVA), and highly substituted hydroxypropyl cellulose (HPC), which are biocompatible materials, were chosen to prepare the microneedles. On the one hand, the specific area of the polymer material is used to increase the drug loading of progesterone, and on the other hand, the study constructs an inclusion carrier–transdermal drug delivery system, which can solve the problem of compatibility between insoluble drugs and water-soluble matrices, and increase the transdermal permeability of progesterone, with broad application prospects.

## 2. Materials and Methods

### 2.1. Materials

Progesterone (98%) was obtained from Hubei Gedian Humanwell Pharmaceutical Co., Ltd. (Ezhou, China). Hydroxypropyl-β-Cyclodextrin (HP-β-CD), polyvinylpyrrolidone (PVP K30), and polyvinyl alcohol (PVA 0588) were provided by Shanghai Macklin Biochemical Co., Ltd. (Shanghai, China). Gelatin was supplied by Sigma Aldrich Co., Ltd. (St. Louis, MO, USA). The hyaluronic acid (molecular weight: 1–10,000) was obtained from Huaxi Biological Co., Ltd. (Jinan, China). Ashland Co., Ltd. (Ashland, KY, USA) provided hydroxypropyl cellulose (HPC, molecular weight: 95,000), and chromatographically pure methanol was procured from Sigma Aldrich Co., Ltd. (St. Louis, MO, USA).

### 2.2. Preparation of the Progesterone Inclusion Complex

The HP-β-CD was dissolved in water and the corresponding molar ratio of progesterone in ethanol (ethanol: water = 1:2 *v/v*, total 3 mL). They were combined, swirled at a rate of 250 r·min^−1^, and heated to the desired temperature. After the completion of the reaction, the ethanol was evaporated under reduced pressure, passed through a 0.45 μm filter membrane to remove the free drug, and then freeze-dried for 24 h. The pre-freezing temperature was −50 °C, and the Vacuum level was 45 mbar. (MODULYOD, Thermo Fisher Co., Ltd., Waltham, MA, USA) [32].

### 2.3. The Single-Factor Experiments

Drug loading and encapsulation rates were used as indicators for comprehensive scoring. The formula is as follows, where L represents the loading rate, E represents the encapsulation rate, and C represents the comprehensive scoring:

$$L=\frac{M\left(\mathrm{Weight}\;\mathrm{of}\;\mathrm{progesterone}\;\mathrm{in}\;\mathrm{the}\;\mathrm{inclusions}\right)}{M\left(\mathrm{Weight}\;\mathrm{of}\;\mathrm{inclusions}\right)}$$

$$E=\frac{M\left(\mathrm{Weight}\;\mathrm{of}\;\mathrm{progesterone}\;\mathrm{in}\;\mathrm{the}\;\mathrm{inclusions}\right)}{M\left(\mathrm{Total}\;\mathrm{input}\;\mathrm{weight}\;\mathrm{of}\;\mathrm{progesterone}\right)}$$

C=L×0.3+E×0.7

Progesterone inclusion complexes were synthesized at 30 °C for 2 h to evaluate the effects of different molar ratios of progesterone and HP-β-CD (1:1, 1:2, 1:4, 1:6, and 1:8) on the encapsulation rate and drug loading of the progesterone inclusion complexes, and screen for the optimal inclusion ratio.

Under a 2 h inclusion period and a 1:1 molar ratio, five inclusion temperatures of 30, 40, 50, 60, and 70 °C were chosen to screen the effects of different temperatures on the progesterone inclusion complex. Subsequently, the optimal inclusion temperature was determined.

To examine the effects of time on the progesterone inclusion complex at a 1:1 molar ratio and a temperature of 30 °C, five inclusion temperatures (2, 4, 6, 8, and 10 h) were selected. The optimal inclusion time was determined.

### 2.4. Central Composite Design (CCD)

The CCD test was used for the accurate screening of prescriptions. CCD tests are a type of response surface test and an optimization approach that takes a system’s reaction as a function of one or more elements and uses graphical tools to illustrate this functional relationship, allowing us to choose the ideal settings in the experimental design through intuitive observation [33]. According to the results of the single-factor experiment, the main research factors were the molar ratio of progesterone and HP-β-CD and temperature.

### 2.5. Optimization and Validation of the Inclusion Process

The CCD experimental data was fitted to predict the optimum preparation conditions, and a composite score was calculated and compared to the predicted values.

### 2.6. Characterization of Progesterone Inclusion Complexes

#### 2.6.1. Infrared Spectra of Progesterone Inclusion Complexes

Appropriate amounts of progesterone, HP-β-CD, a mixture of progesterone and HP-β-CD, and the progesterone HP-β-CD inclusion complex with an appropriate amount of dried potassium bromide powder were ground, pressed, and scanned using infrared spectroscopy (IR Prestige-21420, Shimadzu Co., Ltd., Kyoto, Japan).

#### 2.6.2. Differential Scanning Calorimetry of the Progesterone Inclusion Complex

Suitable amounts of progesterone, HP-β-CD, progesterone–HP-β-CD inclusion complex, and a mixture of progesterone and HP-β-CD (1:2.16) were placed in a container at a temperature rate of 10 °C·min^−1^, a differential heat procedure of 50 V, a temperature rise range of 30–500 °C, and a nitrogen atmosphere (STA 449 f3, NETZSCH, Co., Ltd., Selb, Germany). 

### 2.7. Preparation of Layered Microneedles of the Progesterone Inclusion Complex

When microneedles are inserted into the skin, the drug quickly and effectively enters the body at its tip. Therefore, when preparing drug delivery microneedles, it is vital to consider drug use at the tip of the microneedle. This study investigated the needle tip utilization of different drug prescriptions regarding concentration and solubility. In order to separate the tip of the manufactured microneedles from the backing layer in a closed chamber, the tip and the backing layer were collected separately, 10 mL of methanol was added to each, sonicated for 30 min, and then measured by HPLC analysis to determine the needle tip drug utilization. The following formula was used: Needle tip loading rate (%) = (drug content in microneedle tips/drug content of the entire microneedle) × 100% [34]. The microneedle molds used have a pyramidal needle form, a tip height of 1000 μm, a base edge of 420 × 420 μm, and a number of 400 tips.

### 2.8. Preparation of Microneedles with PVA as the Backing Layer Material

#### 2.8.1. Selection of Needle Tip Materials

Hyaluronic acid (HA), gelatin (GEL), and polyvinylpyrrolidone (PVP) were selected as the primary materials to prepare the microneedle tip, and a polyvinyl alcohol (PVA) solution was used to prepare the backing layer. First, a specific concentration of an aqueous solution of progesterone inclusion compound was prepared by adding different materials to prepare 5% HA (*w*/*v*), 5% GEL (*w*/*v*), and 5% PVP (*w*/*v*) solutions, and a 50% PVA (*w*/*v*) solution in an 80 °C water bath, with water as the solvent. Next, 1 mL of HA, GEL, and PVP needle solutions containing progesterone inclusion compounds were added to the microneedle mold and placed in a vacuum dryer. In the microneedle mold, 1 mL of the tip solution was added and placed in a vacuum dryer in a water bath at 70 °C. The circulation pump was turned on, and the solution was allowed to enter the microneedle mold under negative pressure (0.07 MPa) for 5 min minutes before being dried and demoulded at room temperature to obtain preliminary microneedles, and the residual needle tip solution was absorbed. After drying, 1 g of a 50% PVA (*w*/*v*) solution was added and then placed in a vacuum dryer under negative pressure (0.07 MPa) for 5 min, allowing the solution to enter the microneedle mold and dry off. Next, the microneedle tip was separated from the backing layer, 10 mL of methanol was added, and the solution was taken after 30 min of ultrasonic treatment, and the content measured using HPLC.

#### 2.8.2. Selection of the Needle Tip Concentration

GEL was used as the tip material to screen for the effects of different concentrations on drug distribution in the microneedles. Therefore, different concentrations of GEL needle tip solutions (2.5, 5, 7.5, 10, and 15% (*w*/*v*)) containing the same progesterone inclusion compound were prepared, and a 30% PVA solution was used to prepare the backing layer.

#### 2.8.3. Screening of the PVA Layer Concentration

GEL (7.5% (*w*/*v*)) was used as the tip material to screen the influence of the PVA concentration in the backing layer on drug distribution in the microneedles. Therefore, PVA solutions with different concentrations (10, 20, 30, 40, and 50% (*w*/*v*)) and 7.5% GEL (containing a specific concentration of progesterone inclusion compound) as the tip solution were prepared.

### 2.9. Preparation of Microneedles with HPC as the Backing Layer Material

#### 2.9.1. Selection of Needle Tip Materials

HA, GEL, and PVP were selected as the primary materials to prepare the microneedle tip, and hydroxypropyl cellulose (HPC) solution was used to prepare the backing layer. First, a specific concentration of an aqueous solution of the progesterone inclusion complex was prepared by adding different materials to obtain 5% HA (*w*/*v*), 5% GEL (*w*/*v*), and 5% PVP (*w*/*v*) solutions. Next, HPC solutions of different concentrations were prepared using anhydrous ethanol as the solvent. In the microneedle mold, 1 mL of tip solution was added and placed in a vacuum dryer in a water bath at 70 °C. The circulation pump was turned on, and the solution was allowed to enter the microneedle mold under negative pressure (0.07 MPa) for 5 min before being dried and demoulded at room temperature to obtain preliminary microneedles, and the residual needle tip solution was absorbed. After drying, 2 g of 25% HPC solution was added and centrifuged at 1500 rpm for 5 min to allow for the backing layer solution to enter the microneedle mold, then dried and demolded. Next, the microneedle tip was separated from the backing layer, 10 mL of methanol was added, and the solution was taken after 30 min of ultrasonic treatment, and the content measured using HPLC. In the preparation of microneedles, using the difference in solubility when preparing the backing layer, the HPC solution was introduced into the mold by centrifugation because the HPC solution contained ethanol as the solvent, and the ethanol evaporated too quickly under negative pressure, resulting in an uneven backing layer.

#### 2.9.2. Selection of the Needle Tip Concentration

GEL was used as the tip material to screen for the effects of different concentrations on microneedles’ drug distribution. Approximately 2.5, 5, 7.5, 10, and 15% GEL (*w*/*v*) needle tip solutions containing the same progesterone inclusion compound were prepared, and a 15% HPC solution was used to prepare the backing layer.

#### 2.9.3. Screening of the HPC Layer Concentration

The effect of HPC concentration in the backing layer on the drug distribution in the microneedles was investigated using 15% GEL (*w*/*v*) as the tip material. Approximately 5, 10, 15, 20, and 25% HPC (*w*/*v*) solutions were prepared, and 15% GEL (containing a specific concentration of the progesterone inclusion compound) was used as the tip solution.

### 2.10. Evaluation of the Appearance of the Progesterone Soluble Microneedles

The two dissolving microneedles with the highest tip loading rates were placed under the camera and microscope (Ecliose Ni-U, Nikon Co., Ltd., Tokyo, Japan) for observation and evaluation of their appearance.

### 2.11. Microneedle Mechanical Strength Evaluation of the Progesterone Inclusion Complex

A sealing film puncture test was performed to assess the mechanical strength of the microneedles. Because the skin is elastic, the experiment used ten layers of parafilm sealing film. Two types of microneedles were applied to ten layers of parafilm sealing film (thickness of each layer: 127 μm) with a specific force (approximately 70 N), and the number of microneedles passing through each layer of the sealing film was recorded and measured three times in parallel. The penetration rate was calculated using the following formula: penetration rate% = (number of microneedles penetrating each layer of sealing film/total number of microneedles per piece) × 100%, and the mechanical properties of a self-made microneedle were evaluated.

### 2.12. Penetration Evaluation of Progesterone Inclusion Complex Microneedles

After the rats were anesthetized and killed, their back skin was stripped, and their hair and subcutaneous fat were removed. After rinsing with normal saline, the water was dried using filter paper. Next, the self-made microneedle was pressed on the stripped and depilated rat skin, and the pressed skin was dyed with a trypan blue solution to stain the damaged skin. After removing the excess trypan blue, the penetration effect of the microneedle on the rat skin was observed.

### 2.13. Microneedle In Vitro Release Evaluation of the Progesterone Inclusion Complex

The microneedle was inserted through a sealing film layer to expose and secure the needle tip between the Franz test cell and receiving cell. The transdermal drug diffusion tester was set to 32 °C, the stirrer rotational speed was 200 r·min^−1^, the receiving solution was 20% ethanol in physiological saline, and the sampling point was set (0.25, 0.5, 1, 2, 4, 6, 8, 10, 12, 24, and 48 h); 2 mL of the receiving solution with the same volume and temperature was sampled at a fixed point (at the same time, the receiving solution at the same temperature was supplemented). The samples were diluted with methanol, filtered through a 0.22 µm microporous membrane, and then released in vitro using an HPLC chromatograph.

### 2.14. In Vitro Transdermal Permeation Evaluation of Progesterone Inclusion Complex Microneedles

The skin on the backs of the rats treated with hair removal the previous day was removed after anesthesia and execution; the subcutaneous fat was removed using ophthalmic scissors, rinsed with saline, and blotted with filter paper. The microneedle was applied to the treated rat skin surface with a specific force and fixed to the receiving cell. The transdermal drug diffusion tester was set at 32 °C, the rotational speed was 200 r·min^−1^, and the receiving solution was 20% ethanol in physiological saline. The sampling points were set (0.5, 1, 2, 4, 6, 8, 10, 12, 24, and 48 h), and 2 mL samples were taken at the scheduled time (2 mL of receiving solution at the same temperature was supplemented at the same time). The samples were diluted with methanol, filtered through a 0.22 µm microporous membrane, and then released in vitro using an HPLC chromatograph.

## 3. Results

### 3.1. Single-Factor Experiments

**Figure 1:**
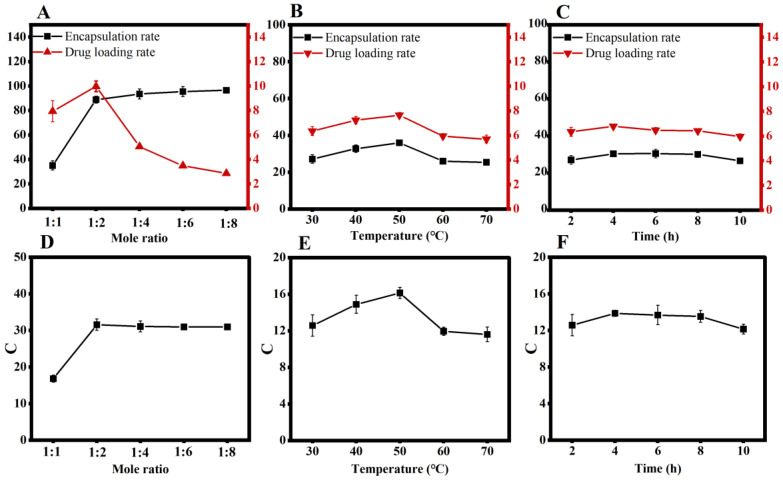
(**A**). Effect of molar ratio change on the encapsulation and drug-loading rate. (**B**). Combined score for different temperatures. (**C**). Effect of temperature change on the encapsulation and drug-loading rate. (**D**). Combined score for different molar ratios. (**E**). Effect of time change on the encapsulation rate and drug-loading rate. (**F**). Combined score for different times.

These results demonstrated the effect of the molar ratio of the progesterone inclusion complex. At a molar ratio of 1:4, the encapsulation rate increased marginally with HP-β-CD. Finally, the loading rate was increased to a maximum of 1:2 by using HP-β-CD (Figure 1A,D). 

The effect of temperature on progesterone inclusion is shown in Figure 1B–D. At 50 °C, the loading and encapsulation rates of progesterone HP-β-CD inclusion were the highest (Figure 1B,E).

The effects of time on progesterone inclusion are shown in Figure 1E,F, which peaked at 4 h, and the encapsulation rate at 6 h (Figure 1C,F).

### 3.2. Central Composite Design

The design and results of the star point response surface experiments are shown in Table 1. The data were analyzed using Design-Expert software, which shows that the model is highly significant and the misfit term is not significant, proving that the model can evaluate the relationship between the molar ratio, temperature, and the overall score well. The results of the ANOVA on the data are shown in Table 2. A (mole ratio), A^2^ (quadratic effect of molarity), and B^2^ (quadratic effect of temperature) had a highly significant effect on the model, and there was no interaction between AB (molarity and temperature).

**Figure 2:**
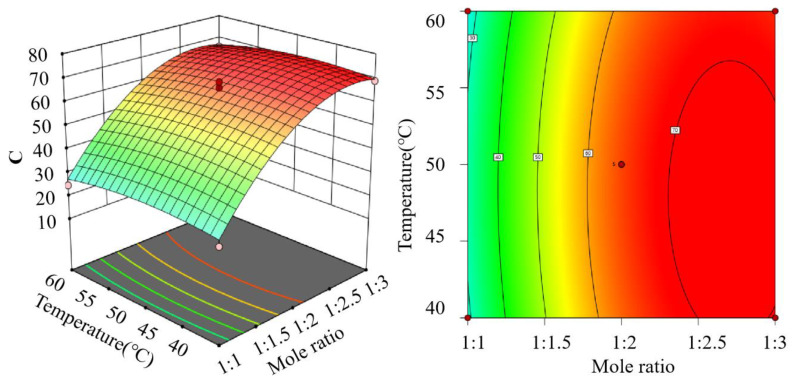
Interaction plot of the molar ratio and temperature with the combined score.

A quadratic regression of the data gave: C = −99.78 + 77.67A + 2.80B − 0.03AB − 14.02A^2^ − 0.03B^2^, *R*^2^ = 0.9888. The simulation picture for this equation is Figure 2.

### 3.3. Optimization and Validation of the Inclusion Process

The quadratic regression equation was solved using the “Design Expert 13” software, and the optimum preparation conditions were obtained with a molar ratio of 1:2.160 and a temperature of 50.137 °C. The optimal preparation conditions were revised to a molar ratio of progesterone to HP-β-CD of 1:2.16, an encapsulation temperature of 50 °C and an encapsulation time of 4 h. The experimental verification of C was 68.31, and the deviation from the predicted value was 0.61%, which was less than 2% and the model prediction was accurate. The progesterone hydroxypropyl-β-cyclodextrin inclusion complex produced under optimal conditions had a drug loading capacity of 9.55 ± 0.21% and an encapsulation rate of 93.49 ± 1.83%. (Table 3).

### 3.4. Characterization of Progesterone Inclusion Complexes

#### 3.4.1. Infrared Spectra of Progesterone Inclusion Complexes

**Figure 3:**
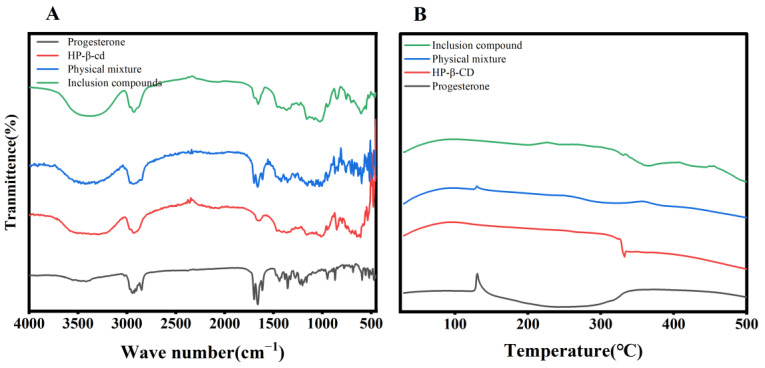
(**A**). Infrared spectra, and (**B**). the DSC result.

The IR spectra of progesterone revealed three absorption peaks at 1698, 1662, and 1617 cm^−1^; 1662 cm^−1^ was attributed to the C=O stretching vibration at the C_3_ position, 1698 cm^−1^ was attributed to the C=O at the C_20_ position, and 16,662 cm^−1^ was attributed to the C=C bond stretching vibration. The peaks at 1698 and 1662 cm^−1^ weakened, and the peak at 1617 cm^−1^ disappeared when progesterone formed an inclusion complex with hydroxypropyl-β-cyclodextrin. In contrast, 1698, 1662, and 1617 cm^−1^ appeared simultaneously in the physical mixture. This suggests that the progesterone was entering the interior of the HP-β-CD cavity when the inclusion complex was formed, rather than simply mixing together. (Figure 3A).

#### 3.4.2. Differential Thermal Analysis of Progesterone Inclusion Compounds

Progesterone exhibited heat uptake, followed by a wide range of minor exothermic phenomena at 130 °C. HP-β-CD exhibited exothermic linearity at approximately 332 °C. The physical mixture of progesterone and HP-β-CD showed significantly less heat uptake at 130 °C and an exothermic peak near 332 °C. The progesterone inclusion complex showed no heat uptake at 130 °C, a reduced exothermic phenomenon, or a prolonged exothermic period at 332 °C. This shows that the progesterone inclusion complex is distinct from progesterone and HP-β-CD, and that progesterone and HP-β-CD are not physically intermingled (Figure 3B).

### 3.5. Progesterone Dissolving Microneedle Loading Measurement

The 15% GEL-5% HPC microneedle had more drug loading in the whole microneedle than the 7.5% GEL-50% PVA microneedle, probably because the higher concentration of GEL, with its greater viscosity, had more of the drug-containing solution adhering to the pores of the microneedle tip during the preparation of the microneedle tip, the results of which are shown in Table 4.

### 3.6. Preparation of Microneedles with PVA as the Backing Layer Material

#### 3.6.1. Screening of the Tip Material

**Figure 4:**
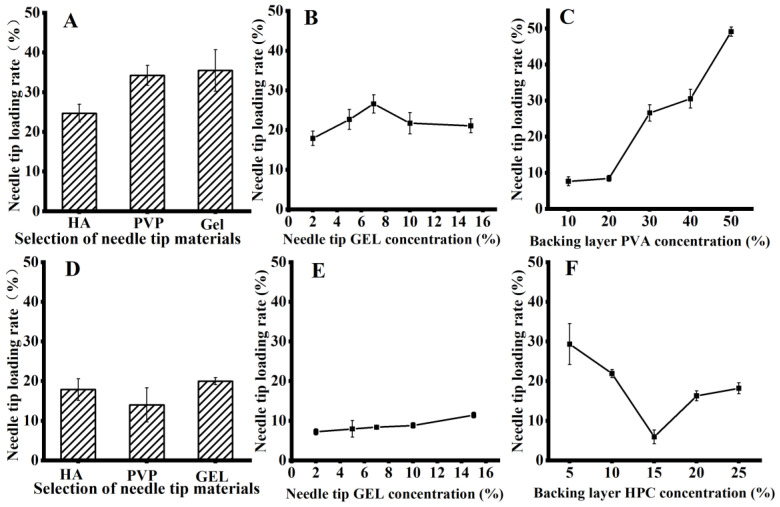
(**A**). Screening of tip materials when using PVA as the backing layer. (**B**). Screening of tip material GEL concentration when using PVA as the backing layer. (**C**). Screening of PVA concentration of backing material when using PVA as the backing layer. (**D**). Screening of tip materials when using HPC as the backing layer. (**E**). Screening of tip material GEL concentration when using HPC as the backing layer. (**F**). Screening of HPC concentration of backing material.

According to the high-performance liquid-phase test results, the drug tip utilization rate in microneedles using GEL as the needle tip material (PVA as the backing layer) was higher than that of PVP and HA materials. The results are shown in Figure 4A.

#### 3.6.2. Screening of the Needle Tip Concentration

Using GEL as the microneedle tip material to explore the effect of the microneedle tip GEL concentration on drug tip utilization, we found that when the tip material was prepared with 7.5% GEL, the drug tip utilization was the highest. The results are shown in Figure 4B.

#### 3.6.3. Screening of the PVA Layer Concentration

When the concentration of PVA in the backing layer was screened, it was found that as the concentration of PVA in the backing layer increased, the drug tip utilization also increased; however, as the concentration of PVA increased, the concentration of the PVA solution also increased. As the difficulty of entering the mold increased, the maximum PVA concentration was 50%. The results are shown in Figure 4C.

### 3.7. Preparation of Microneedles with HPC as Backing Layer Material

#### 3.7.1. Screening of the Needle Tip Materials

When different microneedle tip materials were tested using HPC as the backing layer, microneedles with GEL as the tip material had the highest drug tip utilization rate, followed by those with HA and PVP, which had the lowest. Moreover, GEL and HA were insoluble in anhydrous ethanol; the results are shown in Figure 4D.

#### 3.7.2. Needle Tip Concentration Screening

The needle tip utilization of the drug increases with the GEL concentration; at a 15% GEL concentration that can smoothly enter the mold for preparing microneedles, the needle tip utilization is high, as shown in Figure 4E.

#### 3.7.3. Screening of the HPC Layer Concentration

Using 15% GEL as the material for the preparation of microneedle tips, a study on the effect of HPC concentration on the drug tip utilization rate revealed that the drug tip utilization rate tended to increase and decrease with the increasing HPC concentration. The highest tip utilization rate was observed at 5%, as shown in Figure 4F.

In the preparation of layered microneedles using concentration differences, the prescription with the highest needle-tip loading rate was 7.5% GEL-50% PVA, with a needle-tip loading rate of 49.13 ± 1.29%, and the optimal prescription of 15% GEL–5% HPC microneedle tip utilization rate of 29.31 ± 5.15% was screened using solubility differences.

### 3.8. Evaluation of the Appearance of the Progesterone Soluble Microneedles

**Figure 5:**
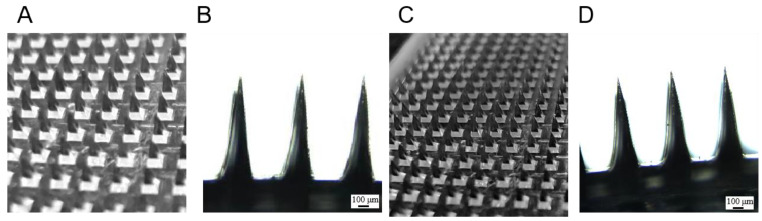
(**A**). 7.5% GEL-50% PVA microneedle camera; (**B**). 7.5% GEL-50% PVA microneedle microscopy (×200); (**C**). 15% GEL-5% HPC microneedle camera; (**D**). 15% GEL-5% HPC microneedle microscope (×200).

The 7.5% GEL-50% PVA and 15% GEL-5% HPC microneedles were observed under the camera and microscope. The prepared microneedles were pyramidal in shape, well formed, and had sharp tips. Both microneedles had similar tip appearances because the materials used to prepare the tips were the same, and the results are shown in Figure 5.

### 3.9. Mechanical Strength Evaluation of Progesterone Microneedles

**Figure 6:**
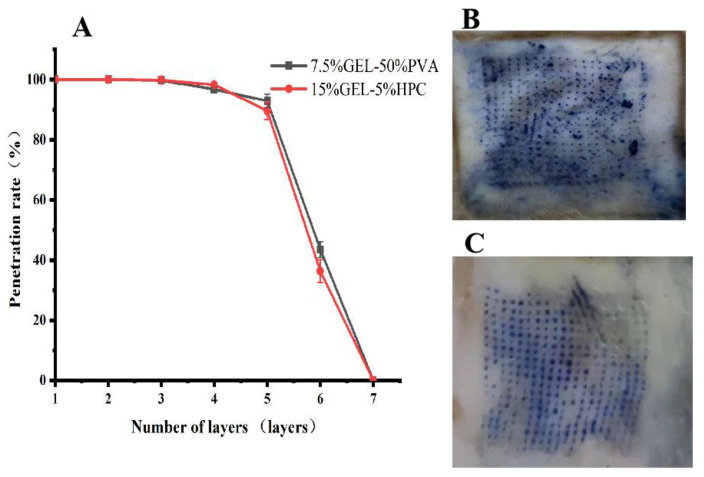
(**A**). Mechanical strength evaluation of progesterone-encapsulated microneedles. (**B**). Penetration evaluation of the 7.5% GEL-50% PVA microneedles. (**C**). Penetration evaluation of the 15% GEL-5% HPC microneedles.

Two different microneedle prescriptions were found to penetrate the six-layer Parafilm sealing film, and the pressed microneedles had an intact needle shape and good mechanical strength. There was no significant difference in the mechanical strength of the two types of microneedles. The results are shown in Figure 6A.

### 3.10. Microneedle Penetration Assessment of the Progesterone Inclusion Compound

Both microneedles penetrated the skin, leaving small, uniform holes in the rat skin through which the drug could bypass the skin barrier and enter the tissue (Figure 6B,C).

### 3.11. Evaluation of In Vitro Release of Progesterone Inclusion Compound Microneedles

**Figure 7:**
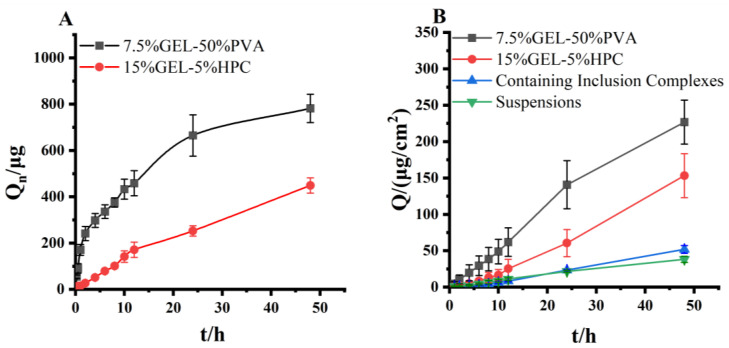
(**A**). In vitro release of progesterone inclusion compound microneedles. (**B**). Outcomes of in vitro transdermal permeation evaluation of progesterone inclusion compound microneedles.

The experimental results showed that the progesterone inclusion microneedles released 781.80 ± 61.14 µg of 7.5% GEL-50% PVA and 448.46 ± 33.08 µg of 15% GEL-5% HPC at 46 h in a 20% ethanol-saline solution. According to the results of the experiments, microneedles with high tip drug utilization have faster in vitro release. The release curves are shown in Figure 7A.

### 3.12. Evaluation of In Vitro Transdermal Permeation Evaluation of Progesterone Inclusion Compound Microneedles

The increased in vitro transdermal permeation of progesterone inclusions compared to a progesterone suspension may be due to the fact that the inclusions increase the solubility of progesterone in water and allow it to cross the skin barrier more easily. The higher in vitro transdermal permeation of the two inclusion microneedles compared to the progesterone suspension and progesterone inclusion suggests that the microneedles can penetrate the cutaneous stratum corneum barrier for effective delivery of progesterone, with the 7.5% GEL-50% PVA microneedle having a higher in vitro transdermal permeation than the 15% GEL-5% HPC microneedle, with the cumulative transdermal permeation of the 7.5% GEL-50% PVA microneedle being 296.89 ± 35.96 μg/cm^2^ compared to 257.88 ± 24.39 μg/cm^2^ for the 15% GEL–5% HPC microneedle, due to the higher tip loading of the 7.5% GEL-50% PVA microneedle (Figure 7B). The transdermal coefficient J was obtained by regressing Q on t. The transdermal coefficients were 4.88 μg/cm^2^/h for 7.5% GEL-50% PVA and 3.27 μg/cm^2^/h for 15% GEL-5% HPC.

## 4. Discussion

Dissolving microneedles are microneedle preparations that are usually prepared using soluble material. After application to the skin, the tip of the microneedle dissolves in the subcutaneous tissue and releases the drug [35]. The dissolving microneedles were prepared using a two-step method. The microneedle tip, which usually contains the drug, was prepared first, and the backing layer of the microneedle was prepared later [36]. The backing layer can be made from a soft material.

An inclusion compound is an organic crystal that typically consists of two parts: one is a compound with a cavity skeleton, and the structure can be other compounds wrapped in this cavity, known as the inclusion agent or body molecule, and the other part of the compound is wrapped in the cavity or pore structure, known as the inclusion agent or guest molecule [37]. For example, hydroxypropyl-β-cyclodextrin (HP-β-CD) introduces a large amount of hydroxypropyl into the chemical structure of β-CD [38]. The introduction of hydroxypropyl partially opens the intramolecular hydrogen bonds of β-CD, improving its water solubility and providing better stability and lower toxicity.

In this study, progesterone microneedles were prepared to effectively penetrate the skin stratum corneum, improve progesterone permeability, and be painless. Progesterone was loaded as an inclusion to increase the water solubility of progesterone and increase the drug loading of soluble microneedles. The molar ratio of progesterone to hydroxypropyl-β-cyclodextrin was 1:2.16, the temperature was 50 °C, and the reaction time was 4 h. The progesterone inclusion complexes had high encapsulation rates and drug-loading capacities of 93.49 ± 1.83% and 9.55 ± 0.21%, respectively.

A low-viscosity PVA was chosen to prepare the backing layer, a commonly used film-forming material, and the prepared film agent had a certain degree of flexibility. Among GEL, HA, and PVP, GEL was selected as the material for microneedle tips, and the 7.5% GEL-50% PVA microneedle was prepared by varying the backing and the tip material concentrations, and the drug-loading rate of the microneedle tip was 49.13 ± 1.29%.

HPC, which has good solubility in both water and ethanol, was selected as the backing layer, and the film formed after drying exhibited good flexibility and toughness. The GEL material proved appropriate for microneedle tip preparation, and a tip utilization rate of 29.31 ± 5.15% was achieved for the 15% GEL-5% HPC microneedle.

Both microneedles exhibited good mechanical strength and pierced the cuticle of the skin during use. The faster release rate of the 7.5% GEL-50% PVA-type microneedle in the in vitro release and in vitro transdermal assays demonstrated that the drug at the tip of the soluble microneedle could be effectively utilized. In contrast, the drug in the backing layer was utilized less. Therefore, the concentration and solubility differences can be exploited to improve the drug-loading rate of the needle tip.

## 5. Conclusions

The microneedles made in this study had a pyramidal shape with good mechanical properties at the needle tip, which could pierce the skin of rats. The backing layer was flexible and flat, fitting the skin. The progesterone inclusion microneedles prepared in the study can penetrate the skin and overcome the stratum corneum barrier, improving the cumulative transdermal permeation of the drug compared to progesterone suspensions and progesterone inclusion compounds.

## Figures and Tables

**Table 1:** Central composite design.

Std.	Run	Mole Ratio (A)	Temperature (B) (°C)	C
13	1	1:2	50	66.08
2	2	1:3	40	68.95
9	3	1:2	50	68.48
3	4	1:1	60	24.64
8	5	1:2	64.14	59.54
11	6	1:2	50	63.50
5	7	1:0.58	50	11.78
1	8	1:1	40	26.36
6	9	1:3.4	50	65.74
7	10	1:2	35.86	62.65
10	11	1:2	50	62.65
12	12	1:2	50	64.65
4	13	1:3	60	65.97

**Table 2:** Variance analysis.

Source	Sum of Squares	F-Value	*p*-Value	
Model	4594.34	123.30	<0.0001	significant
A	3209.25	430.65	<0.0001	significant
B	10.35	1.39	0.2772	
AB	0.3969	0.0533	0.8241	
A^2^	1366.96	183.43	<0.0001	significant
B^2^	56.52	7.58	0.0283	
Residual	52.16			
Lack of Fit	31.02	1.96	0.2626	Not significant

**Table 3:** Prescription optimization.

Number	Loading Rate	Encapsulation Rate	Actual C	Mean C	Predict the C	Deviation (%)
1	9.78	95.35	69.68			
2	9.36	93.44	68.22	68.31	67.90	0.61
3	9.50	91.69	67.03			

**Table 4:** Preparation of progesterone inclusion microneedles.

Type	The Dosage of Needle Tip (μg)	Needapload per Unit Area (μg/cm^2^)	Whole Microneedle Drug Loading Volume (mg)
7.5% GEL-50% PVA	526.11 ± 32.37	233.82 ± 14.39	1.07 ± 0.01
15% GEL-5% HPC	402.86 ± 18.61	179.05 ± 8.27	1.41 ± 0.29

## Data Availability

All data generated or analyzed during this study are included in this article.
